# A Gyroscope Signal Denoising Method Based on Empirical Mode Decomposition and Signal Reconstruction

**DOI:** 10.3390/s19235064

**Published:** 2019-11-20

**Authors:** Chenchen Liu, Zhiqiang Yang, Zhen Shi, Ji Ma, Jian Cao

**Affiliations:** School of Geology Engineering and Geomatics, Chang’an University, Xi’an 710054, China; liuchenchen@chd.edu.cn (C.L.); shizhen@chd.edu.cn (Z.S.); 2015026003@chd.edu.cn (J.M.); 2017126025@chd.edu.cn (J.C.)

**Keywords:** gyroscope, empirical mode decomposition, colored noise, signal denoising, Hausdorff distance, interval threshold

## Abstract

To suppress the random drift error of a gyroscope signal, this paper proposes a novel denoising method, which is based on processing the intrinsic mode functions (IMFs) obtained by empirical mode decomposition (EMD). Considering that a gyroscope signal contains colored noise in addition to Gaussian white noise, fractal Gaussian noise (FGN) was introduced to quantify the noise in the gyroscope data. The proposed denoising method combines the FGN energy model and the modified method of Hausdorff distance (HD) to adaptively divide the IMFs into three categories (pure noise, pure information, and mixed components of noise and information). Then, the information IMFs and the mixed components after thresholding were selected to give the optimal signal reconstruction. Static and dynamic signal tests of the fiber optic gyroscope (FOG) were carried out to illustrate the performance of the proposed method, and compared with other traditional EMD denoising methods, such as the Euclidean norm measure method (EMD-$l_{2}$-norm) and the sliding average filtering method (EMD-SA). The results of the analysis of both the static and dynamic signal tests indicate the effectiveness of the proposed method.

## 1. Introduction

Optical gyroscopes have been widely used in strap-down inertial navigation systems (SINS) [1,2], in which fiber optic gyroscopes (FOGs) have become ideal sensors because of their low cost, light weight, high reliability, easy integration, and high accuracy [3]. FOGs are based on the Sagnac effect, which is mainly composed of a light source, a phase modulator, a fiber coil, a detector, and a coupler, each of which can generate noise [4]. Similar to the core sensor in SINS, the random errors of FOG increase cumulatively with time [5], which further affects the accuracy of the position, velocity, and attitude (PVA). Therefore, suppressing random drift errors is a key issue in gyroscope signal processing.

Two methods are commonly adopted to suppress or eliminate random errors: establishing a random errors model and directly denoising the gyroscope data [6,7]. The former method needs to ensure the accuracy of the random model, and is then combined with a variety of filtering algorithms to supplement the model. However, random errors are susceptible to various uncertainty factors, which have no obvious regularity. Thus, it is difficult to estimate random errors effectively [8]. Since the gyroscope data contains both the actual motion information and the sensor noise, directly denoising gyroscope data can effectively reduce random error. Therefore, the direct denoising method is widely used to reduce random errors of gyroscope sensors [3,6,9,10].

Considering the time-varying, nonlinear, and non-stationary characteristics of gyroscope data, we intend to develop an adaptive denoising method suitable for gyroscope signals. Recently, wavelet transform has been widely used for denoising a non-stationary signal, which performs well at multiple resolutions and in time frequency analysis [10,11,12,13]. It provides a powerful method for noise elimination without establishing an error model. Based on the high-frequency errors and low-frequency signals, the wavelet threshold denoising method [14] has an obvious effect on reducing the random errors of gyroscopes [15]. However, the denoising effect mainly depends on predefined basis functions and the number of layers to be decomposed, which is very unfavorable for adaptability [16,17]. On the other hand, it is worth noting that classic wavelet threshold denoising performed well in removing white Gaussian noise [18]. For the colored noise of the gyroscope data, such as the bias instability, rate random walk, and Markov process, this approach is not ideal for denoising the gyroscope signal [10].

Empirical mode decomposition (EMD) is a time frequency analysis method proposed by Huang et al. in 1998, which can deal well with non-stationary and nonlinear signals [19]. As a completely data-driven method with strong flexibility, EMD can adaptively decompose complex raw signals into intrinsic mode functions (IMFs), ordered from high frequency to low frequency. Apart from its application in the analysis of mechanical vibration signal, meteorology, neurobiology, and images [20,21,22,23,24,25], EMD is well suited to noise estimation and frequency analysis for gyroscope signals in SINS [3,26].

Through decomposition, the EMD can achieve noise reduction by reconstructing the IMFs of information signals and discarding the IMFs of noise [3,9,26]. The statistical characteristics of noise, especially for fractal Gaussian noise (FGN), were analyzed via EMD in [27,28,29]. A denoising scheme was proposed by Ayenu-prah et al. [30] based on the correlation coefficient between the input signal and its IMFs to remove low-order noise IMFs. A cross-correlation de-nosing method was employed to preserve the relevant IMFs [31]. Komaty et al. utilized partial reconstruction based on the similarity measure between the probability density functions (PDFs) of IMFs [32]. However, these partial reconstruction methods with different criteria need to divide IMFs into two groups, namely the noise components and useful information components, while many IMFs contain both [33,34]. Unfortunately, the parts of IMFs containing the main useful information may be lossy because of judgment errors. Instead of discarding the noisy IMFs, low-pass filtering or windowing were performed on the first several noisy IMFs [29,30]. The disadvantage of these methods is that low-order noise IMFs are not completely processed. Boudraa et al. performed threshold filtering on each IMF [35], although this method can reduce noise and causes the loss of useful information. On the other hand, this study is limited to signals contaminated by white Gaussian noise. Inspired by wavelet thresholding, using EMD-based thresholding on the amplitude of IMFs has achieved some preliminary results. An interval thresholding method (EMD-IT) was the first to be introduced. Although thresholding is a more flexible denoising scheme, the wavelet threshold is directly transplanted into the EMD, without further consideration of the noise characteristics of the IMFs. In fact, the data of the inertial sensor contain not only white Gaussian noise but also a large amount of colored noise [10]. In this case, we take the characteristics of the colored noise into account. Given the problems above, an improved EMD denoising method considering FGN for gyroscope signals is proposed.

The remainder of this paper is organized as follows. Section 2 briefly introduces the basic theories of EMD and the error model of a gyroscope associated with FGN; then, our denoising method for gyroscope signals is given. In Section 3, the results and discussions of the proposed method are applied to real data from gyroscopes, and Section 4 offers conclusions.

## 2. Materials and Methods

### 2.1. Basic Theory

#### 2.1.1. Empirical Mode Decomposition

**Figure 1:**
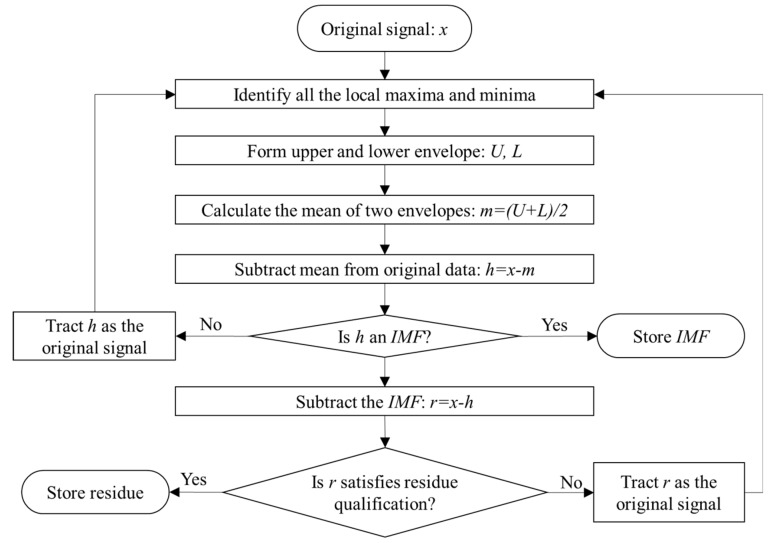
Flow chart of the detailed screening steps of empirical mode decomposition (EMD). Note: IMF = intrinsic mode function.

EMD is a fully data-driven algorithm. EMD can adaptively decompose any signal into several IMFs and a residual [19]. The IMFs and the residual are obtained through a screening process, the detailed steps for which are shown in Figure 1.

There are two judgment processes in Figure 1, which are needed to define the IMFs and the residual. The IMFs have no restrictions on linearity and stationarity, however, two conditions must be satisfied: (1) in the whole signal, the number of extremes must be equal to the number of zero crossings, or at most may differ by one; (2) at any point, the mean value of the envelopes is zero, where the upper envelope is determined by the local maxima and the lower envelope is determined by the local minima. For the residual, this is the stoppage criterion. When the function becomes monotonic or has only one extremum, the screening process finally stops. After decomposition, the signal can be expressed as:

(1) $$x\left(t\right)=\overset{L}{\underset{i=1}{\sum }}h^{\left(i\right)}\left(t\right)+r_{L}\left(t\right)$$

where x(t) is the raw signal; L is the number of IMFs; $h^{\left(i\right)}\left(t\right)\;$ is the *i*-th order IMF; and $r_{L}\left(t\right)$ is the residual.

Through decomposition, all intrinsic mode functions (IMFs) become zero-mean waveforms, and the lower-order IMFs hold more extreme points than that of higher-order IMFs. In other words, the lower-order IMFs capture fast oscillations, while higher-order IMFs typically represent slow oscillations. The noises are mainly contained in the lower order IMFs, while the useful information is contained in the higher order IMFs.

#### 2.1.2. Quantifying the Noise in the Gyroscope Sensor through Fractal Gaussian Noise

Fractal Gaussian noise is essentially a discrete time process, described as an incremental process of fractal Brownian motion (FBM) [36]. FBM is the only self-similar Gaussian process with fixed increments; therefore, its statistical properties are completely dependent on the second moment of parameter H, which is termed the Hurst parameter, satisfying 0 < H < 1 [37]. Typically, fractal Gaussian noise is defined as a zero-mean Gaussian stationary process; its autocorrelation function is:

(2) $$r_{H}\left(\tau \right)=\frac{\sigma^{2}}{2}\left({\left|\tau -1\right|}^{2H}-2{\left|\tau \right|}^{2H}+{\left|\tau +1\right|}^{2H}\right)$$

where $r_{H}\left(\tau \right)$ is an autocorrelation function; τ is the lag length; H is the Hurst parameter; and $\sigma^{2}$ is the variance, which is equal to $r_{H}\left(0\right)$. When τ→∞, we can get:

(3) $$r_{H}\left(\tau \right)\sim H\left(2H-1\right)\;{\left|\tau \right|}^{2H-2\;\;\;\;\;}$$

For H > 0.5, $r_{H}\left(\tau \right)$ decays slowly (long-range dependence) and its value is positive. For H < 0.5, $r_{H}\left(\tau \right)$ decays rapidly (short-range dependence) and its value is negative. Importantly, for H = 0.5, the FGN is the white Gaussian noise:

(4) $$r_{0.5}\left(\tau \right)=\{\begin{array}{c} \sigma^{2}\;\;\;\;\;\;\;\;\;\;\;\;\;\;\tau =0\\ 0\;\;\;\;\;\;\;\;\;\;\;\;\;\;\;\;\;\tau >0 \end{array}$$

Thus, fractal Gaussian noise is a generalization of white Gaussian noise. White Gaussian noise describes uncorrelated error, and fractal Gaussian noise can describe correlated noise according to different H values. This fact extends the applicable range of the error model.

Since the gyroscope sensor contains both uncorrelated noise and colored noise, it is very suitable to use fractal Gaussian noise to construct the error model instead of white Gaussian noise. As the core parameter of FGN, the Hurst parameter should be estimated before the process to construct a noise model containing colored noise. A simple and practical method to estimate the value of the Hurst parameter [27] is power spectral density (PSD). First, take the discrete Fourier transform of Equation (2) to obtain the PSD of FGN:

(5) $$S_{H}\left(f\right)=C\sigma^{2}{\left|e^{i2\pi f}-1\right|}^{2}\overset{\infty }{\underset{k=-\infty }{\sum }}\frac{1}{{\left|f+k\right|}^{2H+1}}$$

where $S_{H}\left(f\right)$ is the PSD of FGN; f is the frequency satisfying |f|≤0.5; and C is the constant. If H≠0.5, f→0, and the PSD of FGN is described as:

(6) $$S_{H}\left(f\right)\sim \;C\sigma^{2}{\left|f\right|}^{1-2H}$$

In the Nyquist frequency band, Equation (6) can be established. This is translated into the double logarithm as:

(7) $$\log S_{H}\left(f\right)\approx \left(1-2H\right)\log\left|f\right|+C$$

A least squares adjustment of Equation (7) is then used to calculate the value of H. Let k be the slope of the fitted line. Then:

(8) $$\hat{H}=\left(1-k\right)/2$$

The Hurst parameter can expose all the information on a gyroscope’s sensor errors. Therefore, after the value of H is estimated, we can quantify the noise in the gyroscope sensor through the FGN.

### 2.2. Proposed Denoising Method

**Figure 2:**
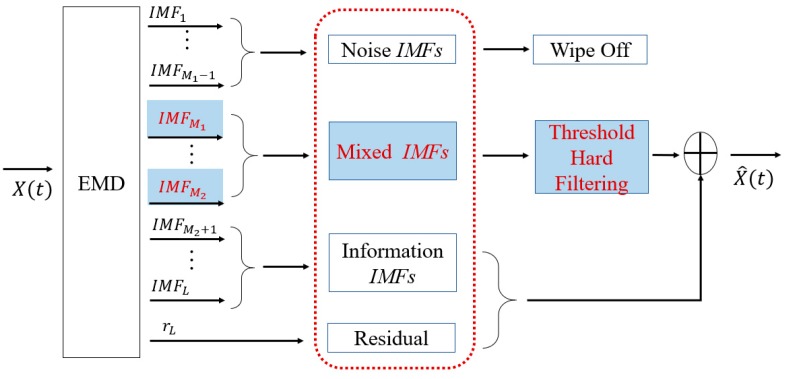
The reconstruction scheme of the optimal signal.

The decomposed IMFs are arranged from high to low according to frequency and amplitude. We divided all IMFs into three categories: noise IMFs, mixed IMFs, and information IMFs. The reconstruction scheme of the optimal signals is shown in Figure 2. The components defined as noise IMFs are discarded, and the mixed IMFs are processed by the hard interval threshold, while the information IMFs and the residual are completely reserved. Based on the above analysis, the denoised signal can be expressed as

(9) $$\tilde{x}\left(t\right)=\overset{M_{2}}{\underset{i=M_{1}}{\sum }}{\tilde{h}}^{\left(i\right)}\left(t\right)+\overset{L}{\underset{i=M_{2}+1}{\sum }}h^{\left(i\right)}\left(t\right)+r_{L}\left(t\right)$$

where $\tilde{x}\left(t\right)$ is the denoised signal; $M_{1}$ is the boundary of the noise IMFs and mixed IMFs, and $M_{2}$ represents the boundary of mixed IMFs and information IMFs; ${\tilde{h}}^{\left(i\right)}\left(t\right)$ is the process of thresholding; $r_{L}\left(t\right)$ is residual. The mixed IMFs and information IMFs can be expressed as $\sum_{i=M_{1}}^{M_{2}}{\tilde{h}}^{\left(i\right)}\left(t\right)$ and $\sum_{i=M_{2}+1}^{L}h^{\left(i\right)}\left(t\right)$, respectively.

In summary, the optimal signals for reconstruction are the mixed IMFs after thresholding, the information IMFs, and the residual. The steps of the proposed denoising method are as follows:

1. Estimate the value of the Hurst parameter (*H*) and quantify the noise through FGN;
2. Implement the EMD algorithm to obtain the IMFs and residual;
3. Determine the pure noise IMFs by comparing the significant difference between the actual and theoretical energy values;
4. Determine the mixed IMFs using an improved Hausdorff distance (*HD*) method;
5. Threshold the mixed IMFs via a hard interval threshold;
6. Reconstruct the optimal signal.

Therefore, the key steps of this method are to classify the IMFs (determine the values of $M_{1}$ and $M_{2}$) and filter the mixed IMF.

#### 2.2.1. Determining the Value of $M_{1}$ and $M_{2}$

To determine the value of $M_{1}$, the energy values of the noise were calculated. Flandrin et al. estimated the empirically observed energy of IMFs in 2004 [27], and proposed that the energy values of pure noise can be estimated as

(10) $${\hat{W}}_{H}\left[1\right]=\overset{N}{\underset{n=1}{\sum }}d_{1,H}^{2}\left[n\right]\;,\;\;\;\;\;\;\;\;k=1$$

(11) $${\hat{W}}_{H}\left[k\right]=C_{H}\rho_{H}^{-2\left(1-H\right)k}\;,\;\;\;\;\;\;\;\;k\geq 2$$

(12) $$C_{H}=\frac{{\hat{W}}_{H}\left[1\right]}{\beta_{H}}$$

(13) $$\rho_{H}=2.01+0.2\left(H-0.5\right)+0.12{\left(H-0.5\right)}^{2}$$

where ${\hat{W}}_{H}\left[k\right]$ is the noise energy estimate of the *k*-th order IMF; *N* is the length of the data; *H* is the Hurst exponent; $d_{1,H}\left[n\right]$ is the first-order IMF sequence; and $\beta_{H}$ is a constant.

For IMF power spectra, the behavior of the first IMF, which has high-pass characteristics, is completely different from that of other modes. For modes *k* = 2 to 6, the power spectra appear to be nearly the same; the spectrum of $IMF_{7}$ gradually evolves from a band-pass to low-pass as the H increases [38]. Based on the characteristics of the spectra, we directly set the first IMF to the pure noise mode, while other noise modes are generated from $IMF_{2}$ to $IMF_{6}$. By comparing the actual energy value and the theoretical energy value of the IMFs, the modes that can match the theoretical value without significantly difference are defined as noise IMFs. Here, we set the maximum difference value of the energy between the theoretical noise and the actual IMF as $M_{1}$.

Next, the Hausdorff distance (*HD*) between the probability density function (pdf) of the input signal and that of each IMF is introduced to determine the value of $M_{2}$. *HD* is a nonlinear operator, which can be used to measure the degree of similarity between two geometric shapes or two sets [39]. The pdf can denote the distribution shape of the signal [40]. By combining the two algorithms, the difference and similarity of the two signals can calculated. The *HD* is defined as follows.

Given two sets $A=\left\{a_{1},\cdots ,a_{M}\right\}$ and $B=\left\{b_{1},\cdots ,b_{N}\right\}$, the distance is defined as:

(14) H(A,B)=max{h(A,B), h(B,A)}

(15) $$h\left(A,B\right)=\underset{a\epsilon A}{\max}\;\underset{b\epsilon B}{\min}\|a-b\|$$

(16) $$h\left(B,A\right)=\underset{b\epsilon B}{\max}\;\underset{a\epsilon A}{\min}\|a-b\|$$

where H(A,B) is the *HD* of *A* and *B.* Here, h(A,B) is the one-way distance from set *A* to set *B*. Similarly, h(B,A) is the one-way distance from set *B* to set A and ‖a−b‖ is the Euclidean distance between the two points.

The probability density functions of the IMFs can be estimated using the kernel density estimator. The similarity measurement between the input signal x(t) and each IMF $h^{i}\left(t\right)$ expressed by the PDF can be defined as follows:

(17) $$L\left(i\right)=\mathrm{HD}\left[pdf\left(x\left(t\right)\right),pdf\left(h^{i}\left(t\right)\right)\right]$$

where L(i) is the similarity measurement and *HD* stands for the calculated distance between the two PDFs estimated by the Hausdorff Distance. $M_{2}$ is the next point of the Hausdorff distance maximum; that is, the first decreasing point, which can be identified by

(18) $$M_{2}=\arg\underset{1\leq i\leq L}{\max}\left[L\left(i\right)\right]+1$$

In order to prevent useful information in the high order IMFs from being discarded, we set $M_{2}$ to be generated in the first half of the IMFs. If there is no decreasing point in the first half of the IMFs, then $M_{2}=L/2$. This improved method can be given by:

(19) $$M_{2}=\min(\arg\underset{1\leq i\leq L}{\max}\left[L\left(i\right)\right]+1,\;L/2)$$

The improved HD method gives a good choice of mixed mode to prevent removing useful information. In this case, the second half of the IMF does not participate in the thresholding.

#### 2.2.2. Thresholding for Mixed IMFs

After determining the $M_{1}$ and $M_{2}$ boundaries of the IMFs, the mixed IMFs can be determined as $IMF_{M_{1}}$ to $IMF_{M_{2}}$, which should be denoised. Inspired by the wavelet threshold, EMD direct thresholding (EMD-DT) is generated [34]. EMD-DT can be modified by hard and soft thresholding [41], which can be expressed as Equations (20) and (21), respectively:

(20) $${\tilde{h}}^{\left(i\right)}\left(t\right)=\{\begin{array}{ll} h^{\left(i\right)}\left(t\right) & \left|h^{\left(i\right)}\left(t\right)\right|>T_{i}\\ 0 & \left|h^{\left(i\right)}\left(t\right)\right|\leq T_{i} \end{array}$$

(21) $${\tilde{h}}^{\left(i\right)}\left(t\right)=\{\begin{array}{ll} sgn\left(h^{\left(i\right)}\left(t\right)\right)\left(\left|h^{\left(i\right)}\left(t\right)\right|-T_{i}\right) & \left|h^{\left(i\right)}\left(t\right)\right|>T_{i}\\ 0 & \left|h^{\left(i\right)}\left(t\right)\right|\leq T_{i} \end{array}$$

where $T_{i}$ is the threshold of the *i*-th IMF. However, the direct hard threshold causes discontinuities, and oscillations are likely to occur. Although the soft threshold is continuous, there is permanent bias from the actual signal after processing. This threshold is a good choice to circumvent permanent bias and overcome discontinuities.

An interval threshold denoising method based on the mode cell filter is introduced. The signal between two adjacent zero-crossings in the IMFs is defined as a mode unit, which can be described as $Z_{j}^{i}=\left[Z_{j}^{i}\;Z_{j+1}^{i}\right]$ in the *i*-th IMF. Thus, the hard threshold is translated into:

(22) $${\tilde{h}}^{\left(i\right)}\left(Z_{j}^{i}\right)=\{\begin{array}{ll} h^{\left(i\right)}\left(Z_{j}^{i}\right) & \left|h^{\left(i\right)}\left(r_{j}^{\left(i\right)}\right)\right|>T_{i}\\ 0 & \left|h^{\left(i\right)}\left(r_{j}^{\left(i\right)}\right)\right|\leq T_{i} \end{array}$$

where $Z_{j}^{i}$ is all the samples from $Z_{j}^{i}\;$ to $Z_{j+1}^{i}$; and $h^{\left(i\right)}\left(r_{j}^{\left(i\right)}\right)$ is the extremum of the mode cell. For the threshold, $T=\sigma \sqrt{2lnN}$ is universal. In this paper, the selection of the threshold takes FGN into account. Here, ${\hat{W}}_{H}\left[1\right]$ and ${\hat{W}}_{H}\left[2\right]$ can be calculated from Equations (10) and (11). Other variations in IMFs can be expressed as:

(23) $$V\left(i^{\prime }\right)=\rho_{H}^{\left(2H-2\right)\left(i^{\prime }-2\right)}{\hat{W}}_{H}\left[2\right]\;\;\;\;\;\;\;\;\;\;\;i^{\prime }>i>2$$

The interval threshold is:

(24) $$T_{i}=\{\begin{array}{ll} \sqrt{{\hat{W}}_{H}\left[i\right]\times 2lnN} & i=1,2\\ \sqrt{V_{\left(i\right)}\times 2lnN} & i=3,\cdots ,L \end{array}$$

**Figure 3:**
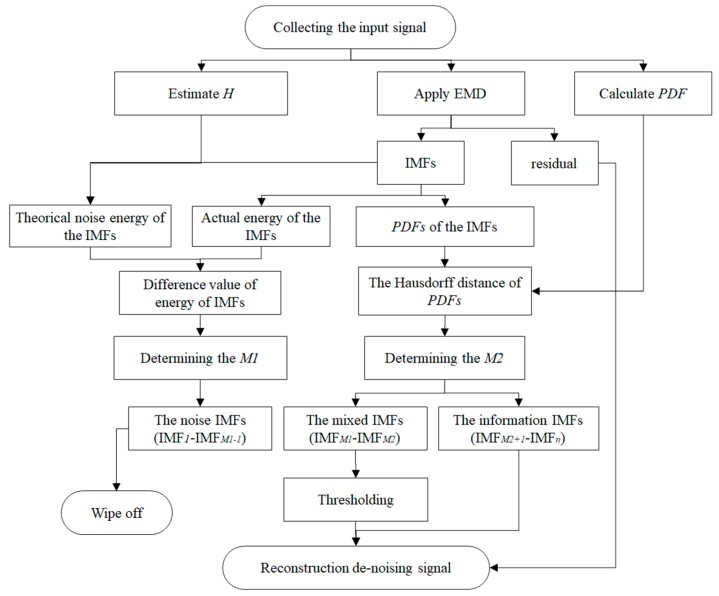
The diagram of the adaptive denoising method. Note: PDF = probability density function.

The framework of our denoising method is shown in Figure 3, where all steps in the framework can be calculated by the above equations.

### 2.3. Experimental Design

**Figure 4:**
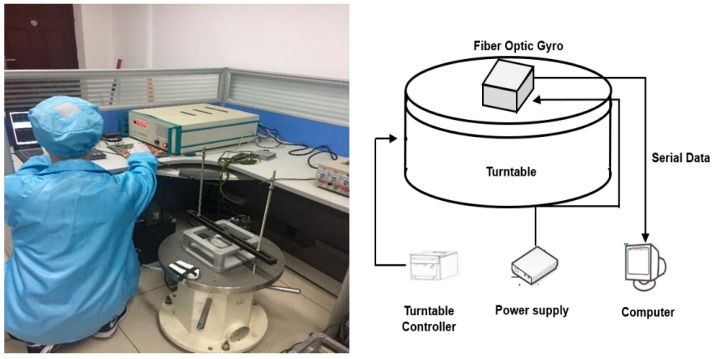
Fiber optic gyroscopes and construction of the turntable test.

The experiments were performed on both static and dynamic test signal, which were collected in a laboratory with a room temperature of 25 °C. The fiber optic gyroscopes (FOG), the construction of the turntable test, and the data transmission equipment are shown in Figure 4. The turntable was made by China National Shipbuilding Corporation; its rotational angular velocity is 0.0001°. The FOG was made by Beijing Aerospace Times Optical-Electronics Technology Co., and is a test product with a zero offset of 0.3 °/h and a sample period of 0.39 ms. The static data collected from the gyroscope remained stationary for more than one hour, and each rotational dynamic datum was collected from the turntable after more than ten minutes. The dynamic test was set to five different uniform rotation speeds: 10 °/s, 20 °/s, 30 °/s, 40 °/s, and 50 °/s, respectively. The FOG and the turntable were restarted for each different test.

Moreover, we compare the denoising effects between the traditional and proposed methods. For the traditional denoising method based on EMD, the partial reconstruction of relevant IMFs (Scheme 2) and the whole reconstruction of the filtered IMFs (Scheme 3) are set. The detail of the experimental schemes are as follows:

**Scheme 1**: Input signal (the actual gyroscope data);

**Scheme 2**: Partial reconstruction of relevant IMFs based on Euclidean norm ($l_{2}$-norm) measure, named EMD-$l_{2}$-norm;

**Scheme 3**: Whole reconstruction of filtered IMFs based on Sliding Average, named EMD-SA;

**Scheme 4**: Proposed method.

To quantitatively illustrate the de-nosing effects of the different methods, we selected the root mean square error (RMSE) as the indicator. The RMSE is calculated as Equation (25), which can reflect the absolute accuracy of the denoising signal,

(25) $$\mathrm{RMSE}=\sqrt{\frac{1}{N}\overset{N}{\underset{t=1}{\sum }}{\left(y\left(t\right)-\tilde{y}\left(t\right)\right)}^{2}}$$

where y(t) and $\tilde{y}\left(t\right)$ are the original signal and denoising signal, respectively; N is the total number of original signals. The smaller the RMSE, the better the performance of the denoising method.

The amplitude spectrum and Allan variance analysis are used to describe the denoising effect, and the values of bias drift, angle random walking (ARW), quantization noise, and RMSE are also calculated for the four schemes.

## 3. Results and Discussions

### 3.1. Static Test Signal

**Figure 5:**
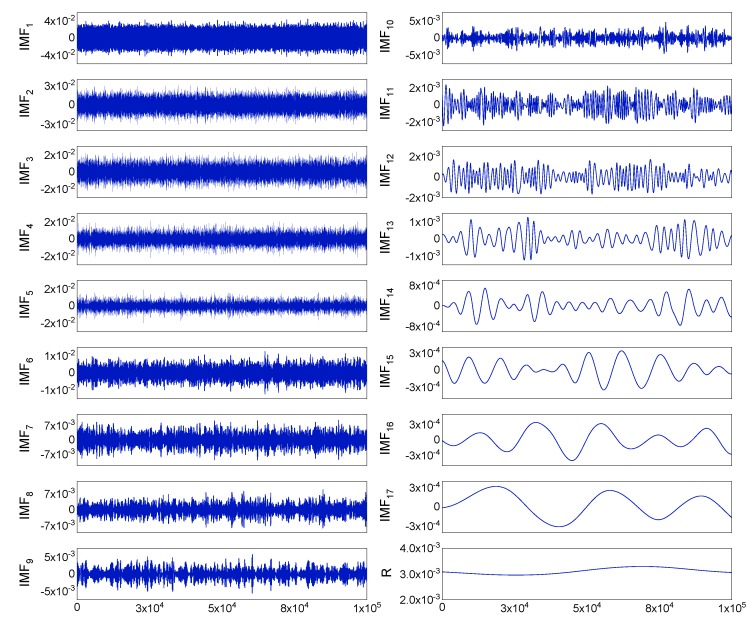
The decomposed intrinsic mode functions (IMFs) and the residual (R) of the noisy signal.

**Figure 6:**
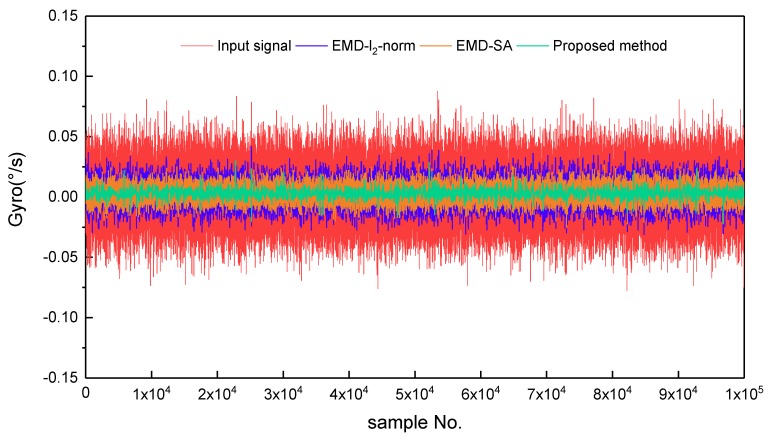
Gyroscope signal and its denoised results for three denoising methods. The red line is the gyroscope signal; the blue, orange, and green lines are the denoising results of Euclidean norm measure method (EMD-$l_{2}$-norm), the sliding average filtering method (EMD-SA), and the proposed method, respectively.

For the real static data, we used a fiber optic gyroscope (FOG) sensor with a zero offset of 0.3 °/h. The sample period was 0.39 ms, and the total number of data samples was *N* = 9262,589. To avoid the influence of human operations of the instrument during the startup and shutdown phases, 100,000 data points in the middle part of the samples were chosen. Using these 100,000 data points and Equation (8), the Hurst exponent was estimated as a value of 0.5167. The data were decomposed into 17 IMFs and one residual by EMD processing, and are shown in Figure 5. After determining the pure noise IMFs, the mixed IMFs, and the information IMFs, by thresholding the mixed IMFs and reconstructing the optimal signal, we obtained the denoising results. Figure 6 shows the static test signal and the denoised signals using the three denoising methods. The proposed method (Scheme 4) offers the best denoising effect, with which the noise is significantly reduced.

**Figure 7:**
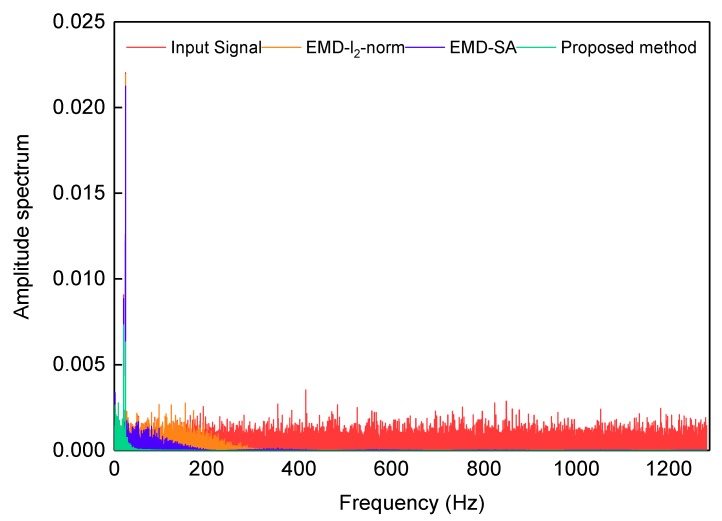
Amplitude spectrum of the signal. The red line is the gyroscope signal; the blue, orange, and green lines are the denoising results of EMD-$l_{2}$-norm, EMD-SA, and the proposed method, respectively.

The amplitude spectra of the signals are obtained by fast Fourier transform (FFT), and are shown in Figure 7. The real test signal of FOG contains significant noise with a high frequency (Scheme 1). The range of amplitude is significantly reduced for high frequencies via the three denoising methods, which means that the higher frequency IMFs of the real test signal have been removed. For the EMD-SA method (Scheme 2), each IMF is filtered to remove a portion of the noise, but the high-frequency noise is retained. The EMD-$l_{2}$-norm (Scheme 3) and the proposed methods (Scheme 4) can deal with high-frequency noise thoroughly, leaving only the low-frequency parts. The proposed denoising method retains the ability to handle low frequency signals.

**Figure 8:**
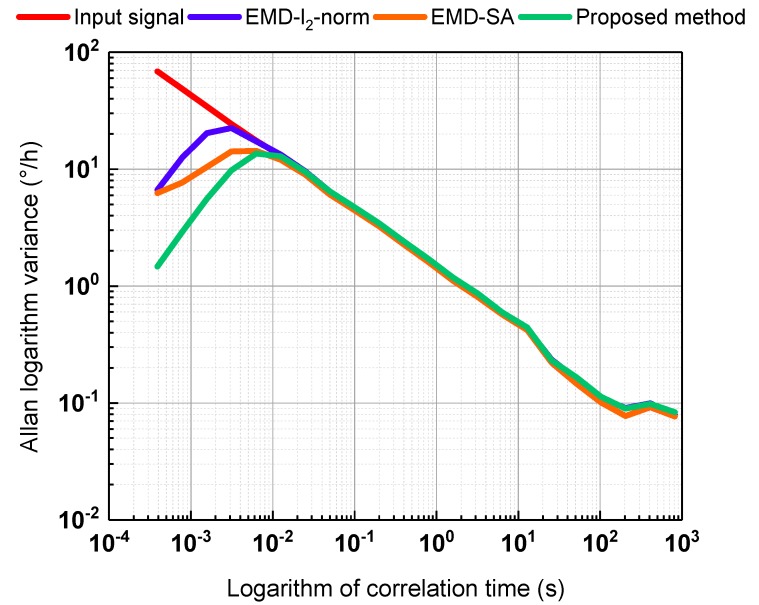
Allan variance analysis of the gyroscope signal and denoised signal. The red line is the gyroscope signal; the blue, orange, and green lines are the denoising results of EMD-$l_{2}$-norm, EMD-SA, and the proposed method, respectively.

Allan variance is recommended by the Institute of Electrical and Electronics Engineers (IEEE) to analyze random gyroscope error. Thus, we adopted Allan variance to further compare the effects of the three denoising methods. The results are shown in Figure 8. The three de-nosing methods can reduce the noise, and the proposed method offers the best performance.

To further accurately describe the denoising effects, we collected a six-hour output signal and calculated the denoising results for different schemes. In Table 1, we can see that the values of bias drift, angle random walking (ARW), quantization noise, and RMSE for the proposed method in this paper are 0.0038, 0.0191, 0.1622, and 0.0045, respectively, which are lower than the values for other denoising methods. Compared with Scheme 1, the proposed method (Scheme 4) improved the denoising effects by 87.7%, 63.4%, 68.4%, and 85.6%, respectively.

### 3.2. Dynamic Test Signal

**Figure 9:**
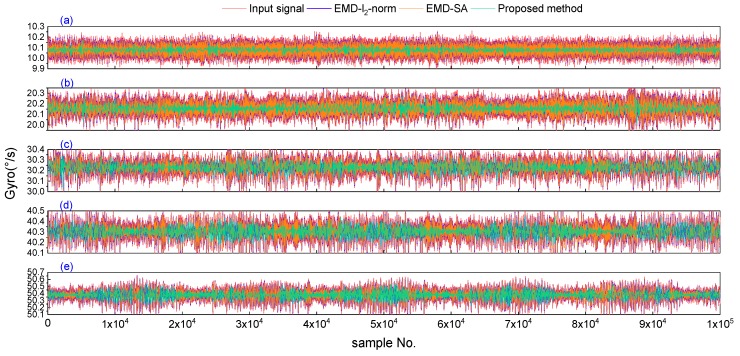
The gyroscope dynamic signal and its denoised results for three denoising methods in the five dynamic tests. The red line is the gyroscope’s dynamic signal; the blue, orange, and green lines are the denoising results of the EMD-*l_2_*-norm, EMD-SA, and the proposed method, respectively. (**a**–**e**) Dynamic gyroscope signals and the denoised results for the three denoising methods, with speeds of 10 °/s, 20 °/s, 30 °/s, 40 °/s, and 50 °/s, respectively.

The FOG in the dynamic test used for data acquisition is the same sensor as the static test; from these tests, five sets of dynamic rotation data were collected. In each dynamic test (the total number of data samples was *N* = 1800,000); we chose 100,000 stable rotation data points in the middle part of the samples for analysis. After the denoising process, the five sets of signals were denoised using the three denoising methods, which are shown in Figure 9. From this figure, we can see that the amplitude of the noise increases with the increase in the rotational speed. The three denoising methods (Schemes 2–4) can reduce the noise in all dynamic data, and the proposed method (Scheme 4) offers the better performance, as the noise can be significantly reduced.

Table 2 shows the results of the dynamic signal with different rotational speeds. In Table 2, we can see that all Hurst values for the dynamic data are less than 0.5. Obviously, the random errors of this gyroscope present a negative correlation in this test. The RMSE and standard deviation (STD) are improved by the three denoising methods (Schemes 2–4), and the proposed method (Scheme 4) has the minimum value. The proposed method improves the RMSE by 4.5 times.

## 4. Conclusions

Traditional denoising methods based on empirical mode decomposition (EMD) are mainly classified into two categories: the partial reconstruction of relevant modes and the whole reconstruction of all filtered modes [26,27]. However, when the signal-to-noise ratio (SNR) of the signal is high, the useful signal is also decomposed into lower-order intrinsic mode functions (IMFs), in which case the useful information may be mistaken for the discarded irrelevant information. To avoid losing useful information, the irrelevant IMFs should be defined as accurately as possible. To this end, we proposed a novel method to denoise the noisy signal; our focus was on discriminating the IMF type (pure noise IMF, mixed IMF, or information IMF). Then, the IMFs in different categories were processed separately.

The proposed method is fully data-driven and adaptive. Firstly, the original signal was decomposed by EMD to obtain IMFs according to the characteristics of the signal itself. Next, in the process of IMF classification and for the interval threshold, no prior parameter settings were required to obtain the optimal signal reconstruction. Then, the values of the threshold and the mode demarcation points required for denoising were also calculated entirely from the IMFs.

This improved method can reduce high frequency noise from a noisy signal, which offers enhanced performance compared to other denoising methods in our experiment. For the real signal test in this paper, the denoising results of the static and dynamic data indicate the effectiveness of the new method, which improves the values of bias drift, angle random walking (ARW), quantization noise, and root mean square error (RMSE) by 87.7%, 63.4%, 68.4%, and 85.6%, respectively. However, the value of ARW ranges from 0.0526 to 0.0192 °/$\sqrt{h}$ and the value of the bias drift ranges from 0.0308 to 0.0038, which is not good compared to state-of-the-art models [1,4]. The FOG we used is not highly accurate. In addition, improved EMD algorithms [42,43] and different thresholding methods for mixed IMFs will be adopted in our next study, to further improve denoising performance.

## Figures and Tables

**Table 1:** Bias drift, angle random walking, quantization noise, and root mean square error (RMSE) for the different schemes.

Scheme	Bias Drift (°/*s*)	Angle Random Walking (°/$\sqrt{h}$)	Quantization Noise (°)	RMSE (°/*s*)
**1**	0.0308	0.0526	0.5130	0.0309
**2**	0.0075	0.0374	0.4820	0.0079
**3**	0.0059	0.0273	0.3404	0.0063
**4**	0.0038	0.0191	0.1622	0.0045

**Table 2:** Comparison of root mean square error (RMSE) and standard deviation (STD) for the denoising results of dynamic signals with different rotational speeds.

Rotational Speed	Indicator	Scheme 1	Scheme 2	Scheme 3	Scheme 4
10 °/*s* (H = 0.2472)	RMSE	0.0951	0.0933	0.0927	0.0892
	STD	0.0537	0.0505	0.0493	0.0177
20 °/*s* (H = 0.3277)	RMSE	0.1686	0.1676	0.1672	0.1580
	STD	0.0685	0.0660	0.0645	0.0276
30 °/*s* (H = 0.3137)	RMSE	0.2396	0.2389	0.2386	0.2321
	STD	0.0686	0.0662	0.0652	0.0392
40 °/*s* (H = 0.2472)	RMSE	0.3144	0.3138	0.3136	0.3069
	STD	0.0763	0.0741	0.0731	0.0450
50 °/*s* (H = 0.3310)	RMSE	0.3901	0.3897	0.3896	0.3809
	STD	0.0860	0.0840	0.0832	0.0500

## References

[1] Dell’Olio F., Tatoli T., Ciminelli C., Armenise M.N. (2014). Recent advances in miniaturized optical gyroscopes. J. Eur. Opt. Soc..

[2] Wu B., Yu Y., Xiong J., Zhang X. (2018). Silicon Integrated Interferometric Optical Gyroscope. Sci. Rep..

[3] Cui B., Chen X. (2015). Improved hybrid filter for fiber optic gyroscope signal denoising based on EMD and forward linear prediction. Sensor. Actuat. A.-Phys..

[4] Ciminelli C., Dell’Olio F., Campanella C.E., Armenise M.N. (2010). Photonic technologies for angular velocity sensing. Adv. Opt. Photonics.

[5] Woodman O.J. (2007). An introduction to inertial navigation.

[6] Yang X.H., Ren J.X., Zhao X.M., Chen R. (2012). MEMS Gyro Signal De-noising Based on Adaptive Stationary Wavelet Threshold. Adv. Mat. Res..

[7] Zhang F. (2013). Modeling Study on Random Error of Fiber Optic Gyro. Appl. Mech. Mater..

[8] Ma J., Yang Z., Shi Z., Zhang X., Liu C. (2019). Application and Optimization of Wavelet Transform Filter for North-Seeking Gyroscope Sensor Exposed to Vibration. Sensors.

[9] Yang G., Liu Y., Wang Y., Zhu Z. (2015). EMD interval thresholding denoising based on similarity measure to select relevant modes. Signal Process..

[10] Gan Y., Sui L., Wu J., Wang B., Zhang Q., Xiao G. (2014). An EMD threshold de-noising method for inertial sensors. Measurement.

[11] Antoniadis A., Bigot J., Sapatinas T. (2001). Wavelet Estimators in Nonparametric Regression: A Comparative Simulation Study. J. Stat. Softw..

[12] Cai T.T., Silverman B.W. (2001). Incorporating Information on Neighbouring Coefficients into Wavelet Estimation. Sankhyā Indian J. Stat. Ser. B.

[13] Bessous N., Zouzou S.E., Bentrah W., Sbaa S., Sahraoui M. (2018). Diagnosis of bearing defects in induction motors using discrete wavelet transform. Int. J. Syst. Assur. Eng. Manag..

[14] Donoho D.L., Johnstone I.M., Kerkyacharian G., Picard D. (1996). Density estimation by wavelet thresholding. Ann. Stat..

[15] Luo H.Z., Lin X.Y., Liu L. Research on GPS/SINS Integrated Navigation System Based on Wavelet Transform. Proceedings of the 7th International Conference on Wireless Communications, Networking and Mobile Computing (WiCOM 2011).

[16] Yan R., Gao R.X., Chen X. (2014). Wavelets for fault diagnosis of rotary machines: A review with applications. Signal Process..

[17] Davari N., Gholami A., Shabani M. (2016). Performance Enhancement of GPS/INS Integrated Navigation System Using Wavelet Based De-noising method. AUT J. Electr. Eng..

[18] Zeng K., Huang J., Dong M. (2014). White Gaussian Noise Energy Estimation and Wavelet Multi-threshold De-noising for Heart Sound Signals. Circ. Syst. Signal Pr..

[19] Huang N.E., Shen Z., Long S.R., Wu M.C., Shih H.H., Zheng Q., Yen N.C., Tung C.C., Liu H.H. (1998). The empirical mode decomposition and the Hilbert spectrum for nonlinear and non-stationary time series analysis. Proc. R. Soc. London A Math. Phys. Eng. sci..

[20] Yu D., Cheng J., Yang Y. (2005). Application of EMD method and Hilbert spectrum to the fault diagnosis of roller bearings. Mech. Syst. Signal Pr..

[21] Guangfen W., Wen A., Fajin G., Zhenan T., Jun Y. The Hilbert-Huang Transform and Its Application in Processing Dynamic Signals of Gas Sensors. Proceedings of the 2009 International Conference on Information Engineering and Computer Science.

[22] Jun H., Zhang Q., Sun G., Yang J.C., Xiong J. (2015). A Vibration Signal Analysis Method based on Enforced De-Noising and Modified EMD. I. J. Signal Process. Image Process. Pattern Recognit..

[23] Deng H., Liu J., Li H. (2009). EMD Based Infrared Image Target Detection Method. J. Infrared Millim. Te..

[24] Wang J., Zhang J., Liu Z. (2008). EMD based multi-scale model for high resolution image fusion. GeoSpat. Inf. Sci..

[25] Rakshit M., Das S. (2018). An efficient ECG denoising methodology using empirical mode decomposition and adaptive switching mean filter. Biomed. Signal Process. Control.

[26] Dang S., Tian W., Qian F. (2011). EMD- and LWT-based stochastic noise eliminating method for fiber optic gyro. Measurement.

[27] Flandrin P., Rilling G., Goncalves P. (2004). Empirical Mode Decomposition as a Filter Bank. IEEE Signal Process. Lett..

[28] Wu Z., Huang N.E. (2004). A study of the characteristics of white noise using the empirical mode decomposition method. Proc. R. Soc. London A Math. Phys. Eng. Sci..

[29] Flandrin P., Goncalves P., Rilling G. (2004). EMD Equivalent Filter Banks, from Interpretation to Applications. Hilbert-Huang Transform and Its Applications.

[30] Ayenu-Prah A.Y., Attoh-Okine N.O. (2010). A criterion for selecting relevant intrinsic mode functions in empirical mode decomposition. Adv.Adapt. Data Anal..

[31] Tang Y.W., Tai C.C., Su C.C., Chen C.Y., Chen J.F. (2010). A correlated empirical mode decomposition method for partial discharge signal denoising. Meas. Sci. Technol..

[32] Komaty A., Boudraa A.O., Augier B., Dare-Emzivat D. (2014). EMD-Based Filtering Using Similarity Measure Between Probability Density Functions of IMFs. IEEE T. Instrum. Meas..

[33] Darong H., Lanyan K., Bo M., Ling Z., Guoxi S. (2018). A New Incipient Fault Diagnosis Method Combining Improved RLS and LMD Algorithm for Rolling Bearings with Strong Background Noise. IEEE Access.

[34] Jiang C., Zhang S.B. (2018). A Novel Adaptively-Robust Strategy Based on the Mahalanobis Distance for GPS/INS Integrated Navigation Systems. Sensors.

[35] Boudraa A.O., Cexus J.C. (2006). Denoising via empirical mode decomposition. Proc. IEEE ISCCSP.

[36] Mandelbrot B.B., Van Ness V.J. (1968). Fractional Brownian Motions, Fractional Noises and Applications. SIAM Rev..

[37] Mandelbrot B.B., Wallis J.R. (1969). Computer Experiments with Fractional Gaussian Noises: Part 1, Mathematical Appendix. Water Resour. Res..

[38] Rilling G., Flandrin P., Goncalves P. Empirical Mode Decomposition, fractional Gaussian noise and Hurst exponent estimation. Proceedings of the IEEE International Conference on Acoustics, Speech, and Signal Processing.

[39] Huttenlocher D.P., Klanderman G.A., Rucklidge W.J. (1993). Comparing Images Using the Hausdorff Distance. IEEE Trans. Pettern Anal. Mach. Intell.

[40] Komaty A., Boudraa A.O., Dare D. EMD-based filtering using the Hausdorff distance. Proceedings of the IEEE International Symposium on Signal Processing and Information Technology (ISSPIT).

[41] Kopsinis Y., McLaughlin S. Empirical mode decomposition based soft-thresholding. Proceedings of the 16th European Signal Processing Conference.

[42] Xi X., Zhang Y., Zhao Y., She Q., Luo Z. (2019). Denoising of surface electromyogram based on complementary ensemble empirical mode decomposition and improved interval thresholding. Rev. Sci. Instrum..

[43] Prosvirin A.E., Islam M., Kim J., Kim J.M. (2018). Rub-Impact Fault Diagnosis Using an Effective IMF Selection Technique in Ensemble Empirical Mode Decomposition and Hybrid Feature Models. Sensors.

